# Supplementary material for: GlycoDraw: a python implementation for generating high-quality glycan figures
Source: Glycobiology. 2023 Jul 27;33(11):927–34. doi: 10.1093/glycob/cwad063 (PMC10859633; doi:10.1093/glycob/cwad063)
Supplement: glycodraw_SI2_cwad063 [file glycodraw_si2_cwad063.pdf]

GlycoDraw: A Python Implementation for Generating High-Quality Glycan Figures

Jon Lundstrøm[1], James Urban[1], Luc Thomès[1], and Daniel Bojar[1,*]

[1]Department of Chemistry and Molecular Biology, University of Gothenburg, Gothenburg, Sweden. Wallenberg Centre for Molecular and Translational Medicine, University of Gothenburg, Gothenburg, Sweden.

[*]Corresponding author: daniel.bojar@gu.se

Supplementary Note 2 – Code examples to generate plots in Figure 3

```
## This code is meant to be run in a Jupyter notebook environment
## install and imports

## If you run this script in a regular Python console, outcomment these lines
!apt install libcairo2-dev
!pip install glycowork[draw]
!pip install candycrunch

## This script was made with/for glycowork version 0.7
import random
import numpy as np
import pandas as pd
import networkx as nx
from CandyCrunch.analysis import *
from glycowork.glycan_data.loader import df_glycan, build_custom_df
from glycowork.motif.analysis import make_heatmap, get_differential_expression,
make_volcano
from glycowork.motif.draw import GlycoDraw, annotate_figure
from glycowork.network.biosynthesis import construct_network, plot_network,
evoprune_network


## simulate data and generate heatmap (Figure 3B)
df_species = build_custom_df(df_glycan.loc[df_glycan.glycan_type == 'O'], kind =
'df_species')
df_species = df_species.loc[df_species.Species == 'Homo_sapiens']

glyc_sia = [k for k in df_species.target.values.tolist() if 'Neu5Ac' in k if 'Fuc' not in k if '?' not
in k if len(k) < 60]
glyc_sia = random.sample(glyc_sia, 5)

glyc_fuc = [k for k in df_species.target.values.tolist() if 'Fuc' in k if 'Neu5Ac' not in k if '?'
not in k if len(k) < 60]
glyc_fuc = random.sample(glyc_fuc, 5)

d = {'target': glyc_sia+glyc_fuc,
     'X1': list(np.random.normal(15, 15, 5)) + list(np.random.normal(95, 15, 5)),
     'X2': list(np.random.normal(15, 15, 5)) + list(np.random.normal(95, 15, 5)),
     'X3': list(np.random.normal(15, 15, 5)) + list(np.random.normal(95, 15, 5)),
     'Y1': list(np.random.normal(95, 15, 5)) + list(np.random.normal(15, 15, 5)),
     'Y2': list(np.random.normal(95, 15, 5)) + list(np.random.normal(15, 15, 5)),
     'Y3': list(np.random.normal(95, 15, 5)) + list(np.random.normal(15, 15, 5))
    }
df_test = pd.DataFrame(data=d)
df_test = df_test.set_index('target')

make_heatmap(df_test.T, filepath = 'heatmap.svg')
annotate_figure('heatmap.svg', filepath = 'heatmap_annot.svg')
```

```
## simulate data and generate volcano plot (Figure 3C)
df_species = build_custom_df(df_glycan.loc[df_glycan.glycan_type == 'O'], kind =
'df_species')
df_species = df_species.loc[df_species.Species == 'Homo_sapiens']

glyc_sia = [k for k in df_species.target.values.tolist() if 'Neu5Ac' in k if 'Fuc' not in k if '?' not
in k]
glyc_sia = random.sample(glyc_sia, 20)

glyc_fuc = [k for k in df_species.target.values.tolist() if 'Fuc' in k if 'Neu5Ac' not in k if '?'
not in k]
glyc_fuc = random.sample(glyc_fuc, 20)

glyc_mixed = [k for k in df_species.target.values.tolist() if '?' not in k]
glyc_mixed = random.sample(glyc_mixed, 10)

d = {'target': glyc_sia+glyc_fuc+glyc_mixed,
     'X1': list(np.random.normal(15, 15, 20)) + list(np.random.normal(95, 15, 20)) +
list(np.random.normal(40, 15, 10)),
     'X2': list(np.random.normal(15, 15, 20)) + list(np.random.normal(95, 15, 20)) +
list(np.random.normal(40, 15, 10)),
     'X3': list(np.random.normal(15, 15, 20)) + list(np.random.normal(95, 15, 20)) +
list(np.random.normal(40, 15, 10)),
     'Y1': list(np.random.normal(95, 15, 20)) + list(np.random.normal(15, 15, 20)) +
list(np.random.normal(40, 15, 10)),
     'Y2': list(np.random.normal(95, 15, 20)) + list(np.random.normal(15, 15, 20)) +
list(np.random.normal(40, 15, 10)),
     'Y3': list(np.random.normal(95, 15, 20)) + list(np.random.normal(15, 15, 20)) +
list(np.random.normal(40, 15, 10))
   }
df_test2 = pd.DataFrame(data=d)

res_de = get_differential_expression(df_test2, group1 = [1,2,3], group2 = [4,5,6])
make_volcano(df_test2, group1 = [1,2,3], group2 = [4,5,6], filepath = 'volcano.svg')
annotate_figure('volcano.svg', filepath = 'volcano_annot.svg', scale_by_DE_res=res_de)


## draw glycan fragments (Figure 3E)

glycan = 'Neu5Gc(a2-3)Gal(b1-3)Gal(b1-4)Glc'
mass_list = [768.19, 575.09, 505.08, 718.17, 487.04, 468.00, 424.03, 342.96, 305.94, 287.98]

glycan_frag = CandyCrumbs(glycan, mass_list, mass_threshold = 1.0)

for mass in mass_list:
  try:
    frag = glycan_frag[mass]['Domon-Costello nomenclatures'][0]
  except:
```

```
  pass
GlycoDraw(domon_costello_to_fragIUPAC(glycan, frag), output = str(mass) + '.pdf')
```
